# In Vitro Cancer Models: A Closer Look at Limitations on Translation

**DOI:** 10.3390/bioengineering9040166

**Published:** 2022-04-07

**Authors:** Nina Antunes, Banani Kundu, Subhas C. Kundu, Rui L. Reis, Vítor Correlo

**Affiliations:** 1 Headquarters of the European Institute of Excellence on Tissue Engineering and Regenerative Medicine, 3Bs—Research Institute on Biomaterials, Biodegradables and Biomimetics, University of Minho, AvePark, Zona Industrial da Gandra, 4805-017 Barco, Portugal; nina.antunes@i3bs.uminho.pt (N.A.); bkundu@i3bs.uminho.pt (B.K.); kundu@i3bs.uminho.pt (S.C.K.); rgreis@i3bs.uminho.pt (R.L.R.); 2 ICVS/3 B’s—PT Government Associate Laboratory, 4710-057 Braga, Portugal

**Keywords:** cancer, 3D cancer models, point-of-care modelling tool, gap analysis, commercialization

## Abstract

In vitro cancer models are envisioned as high-throughput screening platforms for potential new therapeutic discovery and/or validation. They also serve as tools to achieve personalized treatment strategies or real-time monitoring of disease propagation, providing effective treatments to patients. To battle the fatality of metastatic cancers, the development and commercialization of predictive and robust preclinical in vitro cancer models are of urgent need. In the past decades, the translation of cancer research from 2D to 3D platforms and the development of diverse in vitro cancer models have been well elaborated in an enormous number of reviews. However, the meagre clinical success rate of cancer therapeutics urges the critical introspection of currently available preclinical platforms, including patents, to hasten the development of precision medicine and commercialization of in vitro cancer models. Hence, the present article critically reflects the difficulty of translating cancer therapeutics from discovery to adoption and commercialization in the light of in vitro cancer models as predictive tools. The state of the art of in vitro cancer models is discussed first, followed by identifying the limitations of bench-to-bedside transition. This review tries to establish compatibility between the current findings and obstacles and indicates future directions to accelerate the market penetration, considering the niche market.

## 1. Introduction

**Figure 1:**
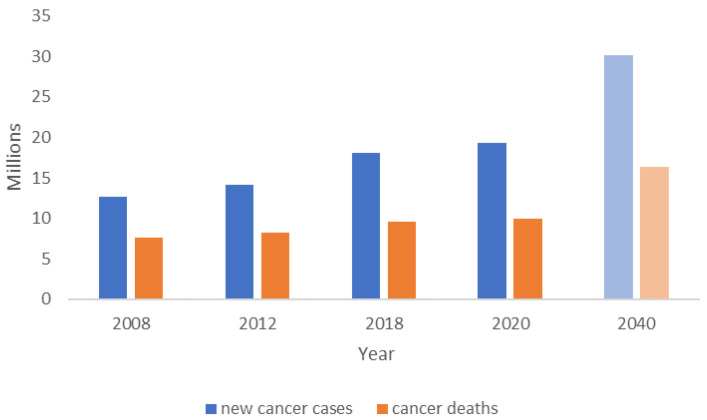
Estimations of new cancer cases and cancer deaths occurred in 2008 [3], 2012 [4], 2018 [5] and 2020 [6] worldwide, including a projection for 2040 (light blue—new cancer cases; light orange—cancer deaths) [2].

Cancer is difficult to treat due to its complexity and diversity—inter- and intra-tumor heterogeneity—and metastasis is the leading cause of death [1]. The number of new cases and deaths is always rising (Figure 1). The future incidence and mortality burden are predicted to increase with a shift in the cancer landscape (more melanoma, pancreatic, and liver cancer cases while fewer breast cancer cases) [2].

**Figure 2:**
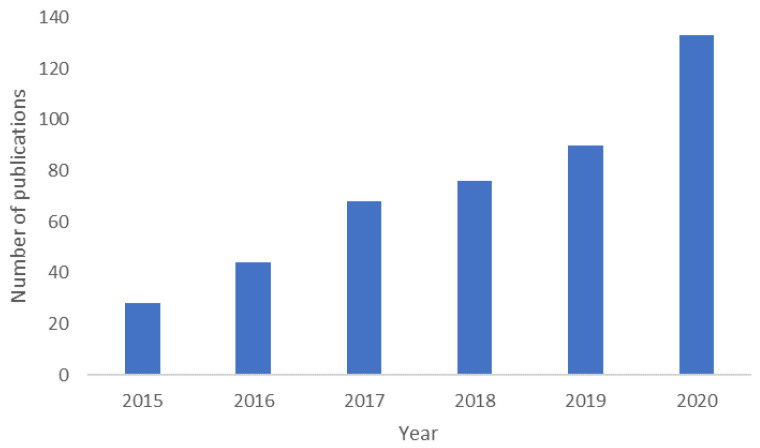
Number of publications in the field of 3D in vitro cancer models in the past five years. The graph is based on the search results using the keywords “3D models” and “cancer” from 2015 to 2020 for documents in English in Scopus. The findings are further narrowed down to those publications which either use 3D in vitro models to study aspects of cancer or describe the development of a new platform.

**Figure 3:**
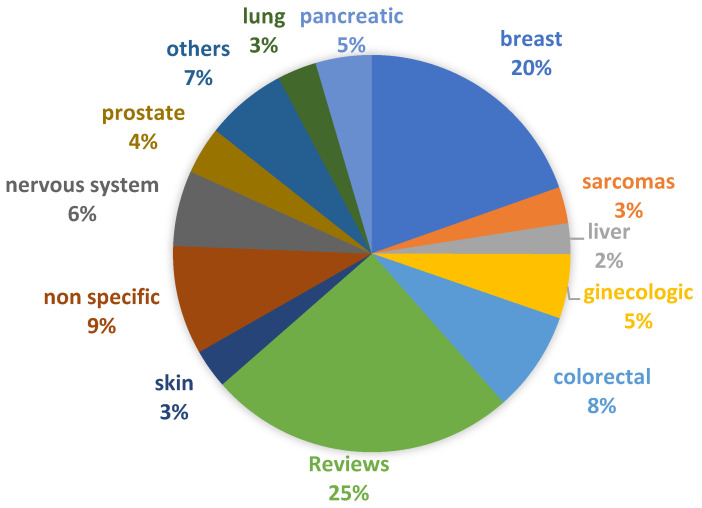
Types of cancer that are represented in 3D models (from the search mentioned in Figure 2). In “others” are included thyroid, renal, gastric, mesothelioma, bladder, head and neck, and blood cancers. “Non-specific” refers to papers that use cells from different cancer types (different cell lines) or do not use cancer cells in the model. “Reviews” also include opinion papers and book chapters.

The field of cancer research is booming with the development of new models interpreting the mechanisms of cancer and identifying early biomarkers, which facilitate the translation of diverse technologies and treatment regimens and is further supported by the increasing number of publications (Figure 2 and Figure 3). The detailed analysis of these publications indicates a paradigm shift in cancer research in the past few decades, from 2D cell culture [7] and animal models [8], including patient-derived xenograft mice (PDX) models [9], to 3D in vitro cancer platforms [10], representing different stages of cancer. However, most of these platforms are derived from established cell lines instead of patient-derived cancer cells. The potentiality of 3D in vitro cancer models in cancer research has been indicated in “The seed and soil theory” proposed by Stephen Paget [11] in 1889. However, it was not until the early 2000s (2002) that the National Cancer Institute [12] launched a US $40 million/year program called “Signatures of Cancer Cell and its Microenvironment”, which enabled 3D cancer modeling. Between 2011 and 2016, the Innovative Medicines Initiative (IMI) founded the PREDECT project to address the failure of the preclinical models involved in predicting drug efficacy. PREDECT was a partnership between academia, SME (Small and Medium Enterprises) and EU pharmaceutical companies, intending to characterize alternative models that represent the complexity and heterogeneity of cancer more realistically [12]. In recent years, the pharma giants such as Pfizer, Merck, Johnson/Johnson, Takeda, Catalent and Roche lead the pharmaceutical drug discoveries by establishing successful collaboration between industries and academic research groups [13]. For instance, Zanubrutinib, marketed by Catalent, is a selective Bruton tyrosine kinase (BTK) inhibitor. It received FDA approval in 2021 for treating adult patients with relapsed or refractory marginal zone lymphoma [14]. The preliminary data of BTK inhibitor, which induced differential cytotoxicity, has been obtained by using in vitro Transwell-based cell migration models [15]. Very recently, it was presented an automated high throughput screening of 150,000 compounds, using as model pancreatic spheroids, which was able to identify leads with potential for further development and application in clinical trials [16].

3D cancer modelling employs the essential components of tissue engineering, such as 3D scaffolding materials, cells, and different signaling molecules, each of which draws the interest from a commercial viewpoint and opens up individual lines of business. The use of the patient’s cells as part of the Human Cancer Models Initiative (HCMI), an international consortium dedicated to creating human tumor-derived culture models, with associated genomic and clinical data, prompts the mass development of personalized cancer treatment platforms. The critical objective of the HCMI consortium is to obtain cancer models that are authenticated, expandable, and conservable for global research [17].

While the forecasts for personalized cancer care are very promising, there is no single route that leads to a complete commercialized cancer model. Each pharmaceutical and biotechnological industry has its own focus to contribute to personalized cancer care, which is summarized in the following section of the review.

## 2. 3D Cancer Models: Product Segments, Commercial Tools, Prototypes, and Patents

Looking back, the modelling of cancer using animals is more than 100 years old. The Ehrlich ascites tumor cells, the spontaneous murine mammary adenocarcinoma cells that rapidly grow in almost all mouse strains, are considered one of the most primitive cancer models [18]. Over the past century, the advancements in cancer cell biology, 3D culture techniques, biomaterials, microfabrication, tissue engineering, and microfluidics have promoted the development of different types of in vitro cancer models. In this section, the current state of the art of commercially available in vitro 3D culture platforms, involved in modelling the different stages of cancer (from initiation, i.e., spheroid formation, migration, invasion, intravasation, extravasation) is summarized. For convenience, they are categorized as follows.

### 2.1. Surfaces and 3D Culture Plates

The simple, ready-to-use, user-friendly, robust platforms piloting the commercial market to generate spheroids are summarized in Table 1.

The major limitations related to spheroids’ development, maintenance and analysis are controlling the size, uniform production, and difficulties in manipulation and handling. These commercially available solutions are already addressing some of these issues.

### 2.2. Scaffolds/Matrices

An alternative approach likely to continue to emerge in cancer modelling is the use of ECM-like elements, such as scaffolds or matrices (e.g., hydrogels, porous sponges, etc.). These ECM components encapsulate the cells, providing cells with structural, mechanical, and physical cues and supporting migration in all three (x, y, and z) directions—closely mimicking the physiological niche. The matrices with variable physiological stiffness ranging from 0.2 to 64 kPa are also available (CytoSoft^®^ Rigidity plates) to mimic the stiffness of cancerous tissue at different disease stages. These scaffolds/matrices are used to investigate the formation of solid tumor-like structures, tumorous growth/proliferation, tumor cell activation, invasion, intravasation, and matrix remodeling. The ECM mimetic components can be synthetic (such as Alvetex^®^, Biogelx™-S, CytoSoft^®^ Rigidity plates) or natural (e.g., Matrigel^®^, PuraMatrix™, HyStem^®^ hydrogels) in origin. The characteristics of different commercially available ECMs, along with their origin, chemical nature, and popular applications, are briefly summarized in Table 2, representing the state of the art of 3D culture.

### 2.3. Patient-Derived and Cell Line-Based Assays/Services, Prototypes

The primary tumor site is preserved in patient-derived models, more specifically, patient-derived tumor xenograft models. PD3D^®^ [31] offers a genomic library of over 200 diverse patient-derived cancer cell strains of 12 different tissue origins for multi-parametric drug response. By incorporating high content imaging, simultaneous recognition of pharmacodynamic biomarkers and anticancer activity can be carried out. Apart from patients’ cells, cell line-based phenotype libraries are also commercially available for therapeutic screening. InSphero’s 3D InSight^TM^ tumor microtissues [32] is a collaborative approach (the service can also be obtained for a fee) to developing advanced 3D tumor/stromal models for therapeutic screening using cell-line derived or PDX-derived tumor microtissues. The OncoPanel™ service of Eurofins Discovery [33] offers a 3D-spheroid based platform with 100 different types of cancer cell lines. However, as an irreversible genetic mutation generates cell lines, this assay platform has limited predictive therapy value in precision medicine. BioIVT’s Tissue Microarrays (TMAs) are another screening tool for identifying new genetic or protein markers for diagnostic purposes, comprising multiple donors (both diseased and healthy). TMA also includes donor and clinical demographics. Fresh human cancer tissue from non-small cell lung cancer is collected and adapted into the 3D culture platform of BioIVT’s, termed the 3D Cancer ORGANDOT^®^ model [34]. Another commercial collection of patient-derived cancer cells is the Kiyatec^®^ ex vivo 3D cell culture platform [35], focusing on ovarian, breast, and glioblastoma. Apart from providing patient-specific physiological tumorous and immune microenvironment to investigate and approve specific cancer therapies, Kiyatec^®^ is also presently involved in the development of 25 different classes of anticancer therapeutic molecules, including checkpoint inhibitors (immune-oncology). Crown Bioscience provides the service and access to the World’s Largest Commercial Collection of Patient-Relevant Models derived from HuPrime^®^ and HuKemia^®^, which are generated from highly characterized PDX models [36]. Their services include tumor growth assays, tumor microarrays, biomarker discoveries, immune-oncology, oncology databases, pharmacological and bioanalytical parameters for high-throughput molecular analysis of cancer tissues and therapeutic development. Besides in vivo models and other in vitro services, Charles River offers pre-defined or customized 3D tumor models for screening. These models can be selected from a library of over 55 Human Cell Line Derived (CDX) models, and 425 patient-derived Xenografts (PDX) models [37]. PharmaLegacy, besides offering a repository of in vivo models, also identifies and validates biomarkers and drug sensitivities through ex vivo assay platforms that employ 3D cultures of patient-derived tumors [38].

In the field of precision oncology, Indivumed is a company that offers a portfolio of biobank products such as a multi-omics cancer database and other services such as analysis of patient-derived tumor tissues to physicians (this service is only available in Germany) [39]. Repositive, a company that joins several contract research organizations (CROs), curates their cancer model data and presents their inventory online, connects these CROSs with the researchers looking for a cancer model [40].

Although established cell lines have been major contributors to cancer research and have been extensively used as cancer models, they are mostly from non-Latino white patients. This causes research disparities, with racial/ethnic minorities having a lack of representation. Patient-derived models, such as PDXs (patient-derived xenografts) and patient derived organoids (PDOs), preserve the molecular features of the original tumor, which makes these models a more accurate route to study tumor development. PDXs and PDOs more accurately translate the therapeutic responses from donors, making these models suitable for biomarker research and drug screening [41].

### 2.4. Microfluidic Platforms

A microfluidic platform is an add-on robotic biofabrication technology to obtain functional tissue-organ constructs, primarily used to investigate cancer cell migration, invasion, intravasation, and extravasation [42]. The SynVivo^®^ [43] offers cell-based, more realistic, microchip services for cell–cell and cell–drug interaction. SynTumor of SynVivo^®^ is an idealized network configuration with 2 μM or 8 μM pore sizes, enabling circulation in the microvasculature and across the vessel walls in the tumor niche (created using tumor cell lines or patient-derived cells). The services of SynVivo^®^ also include target validation, compound screening, biomarker analysis, ADME/Tox and mechanism studies. OrganoPlate^®^ technology developed by Mimetas [44] allows the vascularization of 3D engineered tissue constructs such as organoids, spheroids or tumors in vitro. Tissues (including the PDX tumors) are placed into the chips connected to blood vessels, forming in vitro vascularized 3D construct. The vasculatures are then used to administer the drug in order to ensure that the new anticancer therapeutics have efficient, realistic pharmacokinetics. Further, by incorporating live-cell microscopy (time-lapse-enabled microscope) with the microfluidic platform, the real-time dynamic cellular response within a perfusion-based system can be carried out. This platform is commercially known as CellASIC™ ONIX [45] and is marketed by Merck Millipore.

### 2.5. In Vitro Cancer Models: Patents

The global cancer therapy market has been estimated at USD 13,625,435 million in 2018 and is predicted to be valued at USD 22,070,126 million in 2024 [46]. Factors that drive the market’s growth include Patient Assistance Programs, R&D initiatives from key pharmaceutical industry players, and initiatives increasing cancer awareness.

The report “3D Cell Culture Market by Product, Application, and End User: Global Opportunity Analysis and Industry Forecast, 2020–2027” analyses the market trends and provides future estimations between 2019 and 2027 [47]. In 2019, this market has been evaluated at $1234.86 million. In 2027, estimates predict it will reach $3721 million. In this analysis, cancer research is predicted as the highest growth segment [47].

Patents are a way of protecting intellectual property, with an essential role in translating scientific knowledge into diagnostic means or therapeutic approaches that can help patients [48]. In 1980, the changes in US government policy regarding government-sponsored research’s intellectual property rights marked a new beginning in the commercialization of research results [49]. According to the new policy, the results of federally sponsored research would need to be patented and made available to the private sector for the development of commercial products. Although patents have a vital and unavoidable role in transforming scientific knowledge, they also allow monopolies to operate, blocking products from getting to the market [48]. This particularly concerns the field of unexpansive, affordable health care.

Patenting is a prolonged and extensive process. The pilot programs, such as The Cancer Moonshot Initiative, are created to avoid it; patents4Patients is proposed to expedite cancer research [50]. The Cancer Immunotherapy Pilot Program started in 2016 with the goal of accelerating, without extra fees, patent protection for inventions related to immunotherapy for cancer treatment [50].

**Figure 4:**
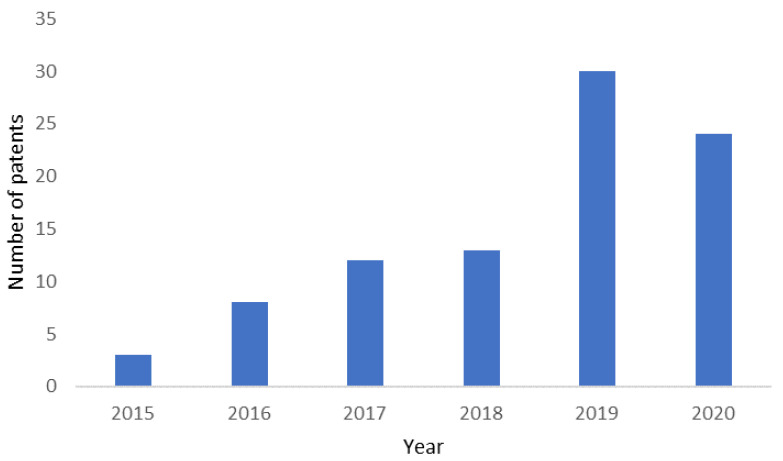
The number of patents issued between 2015 and 2020. Search from the PatentScope database [51] of the World Intellectual Property Organization (WIPO), using the terms “3D cancer model” and the field “front page”.

A simple search on the PatentScope database [51] of the World Intellectual Property Organization (WIPO), using the terms “3D cancer model” and the field “front page”, delivers 90 results, between 2015 and 2020, organized in Figure 4. The increasing trend in the number of patents (materials or methods/prototypes) reflects the rising interests of pharmaceutical companies and biomaterial industries and the expected growth of the therapeutic cancer market in the coming years.

## 3. Gap Analysis: Limitations and Challenges of Existing Models

The last decade has seen an increasing amount of scientific literature—reviews/communications [10,52,53,54,55,56,57,58], book chapters and letters [59,60]. Several authors propose using spheroids as one of the rapid preliminary screening strategies to investigate the potential of anticancer therapeutic molecules [61,62,63,64,65]. Others report new advancement in 3D models, such as in fabrication processes, biomaterial development, and improvements in assay methods and strategies [66,67,68,69,70,71,72,73,74,75,76,77,78,79].

Although over the past decades several scientific and technological advances have been reported in cancer modelling, neo-anticancer therapeutics commercialization does not follow a similar trend [80,81,82]. The number of new therapeutics entering the market per billion US dollars spent on R&D is declining. This trend is called “Eroom’s Law” [80,82]. One possible explanation is the lack of funding to the fundamental research to unwind the unknown biological mechanisms that lead to high failure risk [83]. The current reductionist approaches in cancer models, which include complexity but lack “whole-istic biology” [84], further attributes to it. In 2016, Scannell and Bosley hypothesized that the predictive validity of models has a significant impact on R&D productivity [82]. Hence, the lack of reliable predictive models is a great setback for R&D efficiency [82].

In the early stages of the drug discovery pipeline, when a high number of compounds are screened, simplistic models, such as 2D cell culture, have been used [85]. They are reproducible and less expensive compared to complex models [86]. In the later stages, the use of animal models is required [85,87]. Although 2D cell culture is a convenient model, it does not represent the 3D organization and extracellular matrix (ECM) found in vivo [88]. Animal models also have their limitations, such as the low predictivity of human responses to drugs due to different genomic make up [88]. Also, these models have high costs associated with the animals, and their care and ethical concerns which have been well discussed [87]. For instance, the Transgenic Knockout and Tumor Model Center of Stanford Medicine charges approximately $13,885.49 for a single tumor animal model study involving 30 mice from external investigators [89]. The service includes the injection of tumor cells, measuring the tumor growth 10 times, and collecting tumors at the end of the study. The cost of animal housing is separate. In contrast, Merck’s ready-to-use 3dGROTM Human iPSC Derived Colon Organoids cost only €2220 [90], which is nearly a sixth of the cost of the animal trial.

**Figure 5:**
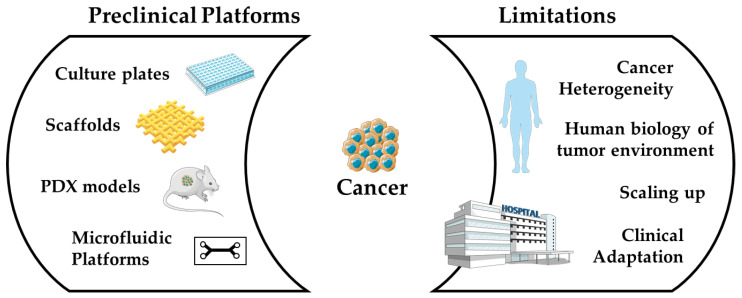
The dual view of cancer models: what already exists (left side) and what is hindering their arrival to the clinics (right side) (This schematic drawing is created using some images from Servier Medical Art (https://smart.servier.com (accessed on 18 September 2020)). Servier Medical Art by Servier is licensed under a Creative Commons Attribution 3.0 Unported License).

The reasons for the decline in neo-therapeutics entering the market have been identified [80,82,83], and pharmaceutical companies are analyzing their projects, trying to find affordable solutions to improve the productivity of R&D [81,91]. To achieve this goal, the Project Data Sphere has been created. This initiative aims to develop a repository of data from cancer trials in order to help improve new trials and accelerate drug discovery [92]. AstraZeneca has come up with five key factors contributing to project failure, calling them the 5 R’s: the right target, the right tissue, the right safety, the right patient, and the right commercial potential [81]. Decision-making and team behavior driven by volume-based goals, instead of emphasizing the understanding of target biology, seem to negatively impact the outcome [81]. As such, a sixth factor arose: the right culture. The failure of neo-anticancer therapeutics in phase 2 of clinical trials is mainly due to a lack of efficacy [93]. In cancer research, target confidence is lower than in other research fields, due to frequent inadequate translation of preclinical screenings to clinics. This phenomenon indicates that cancer models are among the least predictive ones [81,93]. The critical limitations of existing models (Figure 5) are summarized below:

- The hierarchical heterogeneous structure of cancer results in phenotypic and genotypic diversities among the subpopulations of cancer cells. They are not possible to recapitulate in clinical models to date. The reductionist approaches to cancer modelling and the anti-systematic method of therapeutic screening are potent clinical failure recipes [93].
- There are differences between the biology of the model system and the context of the human body. For instance, tumors generally grow faster in laboratory animals or in vitro models than in humans [94].
- The discrepancy between site and stage of the disease in the preclinical model; for example, the subcutaneous tumor xenografts do not mimic the location and setting of the patient’s tumor. Therefore, the experimental therapeutic molecule fails to elicit the desired response at the pre-validated dose concentration [95].
- The inherited constraint of mimicking the advanced disease stage using commonly available cell lines, using more aggressive metastatic variants, such as MDA-MB 231/LM2-4 (triple-negative breast cancer cell line of human into immunodeficient mice (SCID)), to screen the FDA approved anticancer therapeutic Sunitinib, as the therapeutic for advanced metastatic breast cancer, also fails to elicit any response in mono or combination therapy [96].
- The introduction of immune therapy offers a logical approach to overcome the limitations mentioned above and exhibits promising results in treating breast, melanoma, urogenital or non-small cell lung cancers [97]. For instance, Keytruda is a humanized antibody that has received FDA approval as an immune therapeutic agent in the treatment of melanoma, head and neck cancer, and lung cancer patients [98]. However, in these success stories, little consideration is paid to the systematic or local compensatory immune–non-immune response mechanism, the cellular immune composition of site-directed tissues, the oxidation-reduction profile against checkpoint inhibitions, host immune–non-immune response, and adverse side-effects [99,100,101,102,103,104,105]. The systematic insight investigation of the mechanisms of these interdependent pathways and acute inflammatory and effective immune responses must be considered for more effective cancer immune therapy.
- A closer examination of detailed data spanning several decades reveals that persistent injuries, chronic infections, or inflammations cause genetic changes at site-specific tissues, increasing the risk of cancer, particularly in the elderly [102].

In 2020, the cancer burden was estimated at 2.7 million new cases and 1.3 million deaths [106]. However, there is still a lack of recreation of endless genetic mutations and chaotic molecular involvement during disease progression in in vitro tumor models. The high failure rate (~90% ± 5) of cancer chemotherapies, including site-specific targeted therapy (such as missile therapy) or molecular targeting drugs (e.g., inhibitors of growth factor receptors or enzymes) is attributed to the highly reductionist in vitro models [97]. More realistic models that consider factors such as age, disease, or immune-compromised conditions of patients and complex immune-signaling pathways specific to patient are required. The cost–benefit ratio of the current therapies, the scaling up of some of the available novel modelling approaches, the ready adaptation of complex lab-techniques in clinical practice and the need of high-skilled researchers or technicians to operate the high throughput platforms are the other critical restraints [60,107].

The commercial application of human tissue-based models is further limited due to the collection and maintenance of the tissues and access to clinical metadata. Precision-cut tumor slices have the potential to represent the native tumor complexity and heterogeneity, allowing researchers to study the cells in their microenvironment [93]. However, post-processing of slices is critical and restricted to a specific laboratory/system, which causes difficulties in reproducibility [93]. For 3D in vitro models to be integrated into existing workflows, they must be low-cost, rapid, and robust in terms of translation into clinical context [60]. However, it is not easy to standardize the use of these models, since their production requires multidisciplinary approaches that are expensive too [60].

It is possible to incorporate further complexity into the available cancer models, resulting in difficulties in throughput and interpretation [81]. To improve 3D models and R&D productivity, the cost, the throughput ability, and the overall convenience of the model should be weighed against the predictive value of the model. Should we favor one side over the other or look for a balance? A balance should be the answer, but the cost can be overlooked for the precision models for those who can afford them. For this reason, the models showing promising results or potential should be further tested, characterized, and validated academically and industrially.

The incorporation of 3D models into the drug development pipeline has the potential to deliver more translatable data to the clinic and reduce the number of animals used [85]. AstraZeneca and Genentech published a comprehensive study with hepatic spheroids that supports their value for hepatotoxicity risk assessment in drug discovery [108].

Although these models are potentially more expensive than 2D culture, depending on the procedures and equipment used, and have lower throughput, they can be of major importance if used in the target validation phase, increasing target confidence [85].

## 4. Concluding Remarks and Future Perspectives

The growing body of evidence reflects the necessity of more translational interpreting models in immuno- and pharmaco-oncology. Cancer models are continuously evolving and are extended to new stages of therapeutic development, including optimization of therapeutic structures, toxicity and tolerability and precision personalized medicine. Today, choosing an appropriate model that best imitates the given tumor entity is a significant challenge for researchers. In each cancer type, the organization of the cytoskeleton, the mechanism of hypoxia and senescence vary. Cancer is so heterogenous that even within a single cancer type, it varies from individual to individual. This situation makes it critical to model autochthonously. Hence, specific approaches or agents and special techniques are used, such as tumor neo-antigens, genomic sequences of patient and tumor, different omics methods and patient-derived (PDX) xenografts. The large amount of data thus obtained from this personalized modelling is used to establish a pathway to target individual tumors using new bioinformatic tools, named “data science” or “big data” [109].

Advances in 3D culture, tissue engineering, and microfluidic led to the development of cancer-on-a-chip platforms, which are further integrated with artificial intelligence for more significant drug screening [110]. Pathal et al. (2018) indicate that a machine learning algorithm effectively predicts a tumultuous behavior response depending on the system’s past observation. These studies are performed on closed-loop intelligent operation systems.

In the twenty-first century, another phenomenon of cancer that needs to be taken into serious consideration during modelling is cellular senescence and dormancy in cancer progression and therapy resistance. Senescent cells are a part of the cancerous cellular stroma and are often spared by chemotherapeutic agents. The senescent cells then release cytokines or membrane-bound vesicles, known as secretomes, that induce cancerous growth in the neighborhood. Hence, the senescence-associated tissue microenvironment needs to be considered during modelling.

The next-generation platform or model of tumor biology, target discovery, and therapeutic validation platform can be obtained by combining transformative technologies such as genetic engineering (CRISPR/Cas9), single-cell genomics, transplantation model (such as PDX).

One model alone mimicking all the aspects of cancer would probably be impractical. More complex models, able to simulate a specific aspect of the disease, would be of major help. These kinds of models, more representative of the disease in the human context, using patient tissue samples or cells, would contribute to precision (personalized) medicine, indicating the right patients for a specific drug [85].

Lastly, the great improvement in “brute force” efficiency, characterized by high throughput, reproducible and automatized methods, doesn’t contribute to cancer drug discovery [82]. Due to the immense complexity of this illness, a shift to a different approach should be made. Cancer may be better represented by a lower throughput platform delivering higher detail (information) [111], which means prioritizing the predictive validity of the model over the scaling up ability or reduced costs [82].

## Figures and Tables

**Table 1:** Different types of 3D culture plates and surfaces commercially available.

Commercial Products	Marketed by	Features	Limitations	References
AggreWell™	STEMCELL™ Technologies	- Comprises a standardized array of microwells per well; - Rapid and uniform formation of spheroids.	- Absence of cell fate modulatory ECM; - Inappropriate to obtain the insight of biophysical cues in pathophysiologic studies; - Migration, intravasation, and extravasation studies are not possible; - Due to the absence of ECM, false drug concentrations are obtained which are not relevant in clinical practice.	[19]
Corning^®^ Spheroid Microplates	Corning^®^	- Allow the fluorescent or luminescent investigation of spheroids in situ, within the same plate.		[20]
CELLSTAR^®^ Cell-Repellent Surface	Greiner Bio-One	- Prevents cell attachment on the surface while promoting the cell–cell aggregation and spheroid formation.		[21]
NanoShuttle™-PL		- The cells are magnetized using magnetic nanoparticles and the spheroids are achieved by magnetic forces, either by levitation or bioprinting.		
Lipidure^®^-COAT plates	AMS Biotechnology	- Support the formation of the spheroid, embryoid body and organoid culture.		[22]

**Table 2:** Different types of commercially available scaffolds/matrices for 3D cell cultures.

Commercial Products	Marketed by	Features	References
Alvetex^®^	AMS Biotechnology	- A synthetic scaffold for 3D cell culture; - Available as multi-well plate and inserts.	[23]
Biogelx™-S	BIOGELX™	- A synthetic peptide that readily forms hydrogel with a nanofibrous network; - Offers excellent printability with cell viability.	[24]
BiogelxTM-RGD, BiogelxTM-IKVAV, BiogelxTM-YIGSR and BiogelxTM-GFOGER		- Biomimetic ECM protein conjugates with Biogelx™-S for tissue-specific applications.	
Matrigel^®^ and PuraMatrix™	Corning^®^	- Matrigel^®^ is a gelatinous protein mixture secreted by Engelbreth-Holm-Swarm mouse sarcoma cells; - A natural ECM-derived matrix of amino acids (1% *w*/*v*) and 99% water, used to create defined 3D micro-environments.	[20]
CytoSoft^®^ Rigidity plates	Advanced BioMatrix	- A biocompatible silicone-coated plate with variable stiffness (0.2–64 kPa).	[25]
HyStem^®^	Sigma-Aldrich^®^	- A semi-synthetic 3D hydrogel of chemically synthesized hyaluronic acid.	[26]
MaxGel™		- Human basement membrane extracts containing ECM components including collagens, laminin, fibronectin, tenascin, elastin, a number of proteoglycans and glycosaminoglycans.	[27]
TrueGel3D™		- A biochemically defined hydrogel obtained by reaction between polymers with crosslinkers. In contrast to other commercially available matrices, it lacks any component/extract of animal origin.	[28]
Millicoat™		- Coated strips to promote cell adhesion (e.g., vitronectin, collagen type I).	[29]
MAPTrix™	Kollodis BioSciences, Inc.	- A highly controlled 2D extracellular matrix of recombinant Mussel Adhesive Protein; - Can readily be used to coat the standard surfaces.	[30]

## Data Availability

Not Applicable.
